# Enhancing proteotoxic stress as an anticancer strategy

**DOI:** 10.18632/oncotarget.268

**Published:** 2011-04-27

**Authors:** Steven Grant

**Affiliations:** Division of Hematology/Oncology, Department of Internal Medicine, Goodwin Research Laboratory, Richmond, VA

Transformed cells are known to differ from their normal counterparts in a wide variety of ways, including their increased reliance on aerobic glycolysis, defects in DNA damage checkpoint controls, diminished reliance on growth factors for survival, dysregulation of cell cycle control mechanisms, and propensity for dissemination beyond their normal environment, among numerous others. In 2000, Weinberg described six essential “hallmarks of cancer”, providing a theoretical framework for exploiting the transformed state from a therapeutic standpoint [1]. In 2009, Elledge expanded this list to 12 characteristics, including several described as “orthogonal” elements [2]. These processes were invoked to explain the paradoxical observation that certain oncogenes, such as c-myc, while conferring a proliferation advantage on transformed cells, may nevertheless exert pro-apoptotic activities. Consequently, a second aberration (i.e., up-regulation of an anti-apoptotic protein such as Bcl-2) may allow cells to escape the otherwise lethal effects of c-myc dysregulation, and in so doing, cooperate in transformation [3].

Another important “orthogonal” characteristic of transformed cells is their capacity to withstand the accumulation of un- or misfolded proteins, referred to as proteotoxic stress. Neoplastic cells in general exhibit increased protein turnover, and certain tumors e.g., multiple myeloma, have extremely high turnover rates. Ordinarily, such proteins are dealt with by ubiquitination and proteasomal degradation by the ubiquitin-proteasome system (UPS), and this process is facilitated by the induction of a variety of protein chaperones, including members of the heat shock protein family (i.e., Hsp90 and Hsp70) [4]. Increased accumulation of misfolded proteins in the endoplasmic reticulum (ER) can also lead to another form of proteotoxic stress referred to as ER stress. In this case, the unfolded protein response (UPR) consists of various compensatory events, including increased synthesis of ER chaperone proteins, shut-down of protein synthesis (i.e., by PERK/eIF2α), and accelerated protein degradation (ERAD) [5]. Various forms of the proteotoxic stress response can play cytoprotective roles at early intervals, but when the protein load exceeds a certain threshold, they can instead contribute to cellular demise [6].

The increased reliance of transformed cells on systems that ameliorate the deleterious effects of proteotoxic stress has stimulated the development of multiple strategies and agents specifically designed to disable these mechanisms. For example, intense efforts have been directed at developing inhibitors of Hsp90, and several such agents i.e., geldanamycin and more current derivatives such as DMAG, have now entered the clinical arena [7]. Furthermore, the observation that Hsp90 antagonists stimulate the HSF1-dependent induction of Hsp70, and that the latter protein can protect transformed cells from Hsp90 inhibitor-mediated lethality, has prompted the development of Hsp70 antagonists, to be used either alone or possibly in combination with Hsp90 inhibitors [8]. However, the greatest success to date with this group of compounds is that of inhibitors of the 26S proteasome such as bortezomib, which among numerous actions, block protein degradation and in so doing, promote the accumulation of misfolded proteins [9]. Notably, bortezomib has been approved for the treatment of refractory mantle cell lymphoma, as well as relapsed multiple myeloma, a disease characterized by pronounced protein turnover.

Not surprisingly, attempts to combine these strategies in the hope of exceeding the proteotoxic stress threshold and triggering cell death have attracted considerable attention. For example, preclinical studies have shown that simultaneously disrupting Hsp90 function (i.e., with Hsp90 antagonists) and interfering with protein degradation (i.e., by proteasome inhibitors) markedly increases transformed cell death [10], and attempts to translate this strategy into the clinic are currently underway. Similarly, evidence that Hsp70 induction can compensate for inhibition of Hsp90 function has prompted a strategy combining Hsp90 and Hsp70 inhibitors, and this approach has also been found to potentiate neoplastic cell death [8]. In this context, interest has recently focused on HDAC inhibitors as potential modulators of the proteotoxic stress response. For example, it has been shown that inhibition of HDAC6 leads to disruption of the dynein motor responsible for the normal function of aggresomes, which are intimately involved in regulating the proper disposition and subsequent elimination of misfolded proteins [11]. The ability of pan-HDAC inhibitors, which target HDAC6, to disrupt aggresome function has been invoked to explain their potentiation of the antitumor activity of bortezomib i.e., in multiple myeloma cells [12]. In some cells e.g., mantle cell lymphoma cells, this interaction may also involve shifting of the ER stress response from a cytoprotective to a pro-apoptotic process [13].

To date, most approaches attempting to exploit proteotoxic stress from a therapeutic standpoint have focused on combining agents that disable different components of the proteotoxic stress response e.g., chaperone proteins and proteasome function. However, results of a study by Neznanov et al., recently reported in Oncotarget [14] suggest a fundamentally different approach to this problem. Neznanov and colleagues employed the proteasome inhibitor bortezomib to enhance proteotoxic stress of transformed cells subjected to interventions that by themselves increased the cell's burden of misfolded proteins i.e., hyperthermia or the antibiotic puromycin, which causes premature termination of translation leading to the accumulation of misfolded proteins. These investigators found that combined treatment of transformed cells with hyperthermia or puromycin with bortezomib, at exposures that were minimally toxic individually, resulted in a marked increase in protein ubiquitination and cell death. Notably, lethality occurred despite the marked induction of HSF-1-mediated induction of the cytoprotective chaperone protein Hsp70. In addition, while intact p53 function was not required for cell death induced by this strategy, its presence resulted in an increase in lethality. Importantly, the combination of puromycin and bortezomib resulted in enhanced antitumor activity in a murine xenograft model, suggesting that this phenomenon is not restricted to the *in vitro* setting.

The results of this study have potentially important implications, including those that are translational in nature. Currently, considerable interest has focused on targeting those pathways to which transformed cells are either addicted [15] and/or uniquely reliant due to enhanced tumor cell requirements e.g., handling of increased protein turnover or DNA damage [16]. However, it has become increasingly apparent that with rare exceptions, interruption of a single pathway or process is unlikely to have a major benefit in of itself; instead, interruption of multiple complementary pathways may be necessary [17]. In the case of proteotoxic stress, past and current approaches have understandably involved the rational combination agents that disrupt various cellular mechanisms designed to cope with this problem e.g., proteasome and chaperone protein antagonists. It is presumed that the inherent reliance of tumor cells on such protein disposal mechanisms should be sufficient to ensure adequate antitumor activity of these strategies. The implications of the report by Neznanov et al., are that this assumption may not be justified i.e., simply disrupting the proteotoxic stress response at multiple sites may not be sufficient for significant therapeutic benefit, even in those transformed cells known exhibit increased protein turnover. Instead, interventions that increase the cell's burden of misfolded proteins, including hyperthermia or disruptors of translation, may represent superior candidates for combination with proteasome inhibitors (or possibly other agents that interfere with protein disposition) in the clinical setting. In this context, a variety of agents known to block protein translation (e.g., ribavarin, sorafenib) deserve scrutiny [18, 19]. Questions remaining to be addressed include determining whether such strategies do in fact selectively target transformed cells, assessing whether the benefit of these strategies will be restricted to neoplastic cells characterized by high protein turnover, and identifying optimal regimens combining agents that promote proteotoxic stress with those that disrupt protein disposition. It is anticipated that answers to these questions may be forthcoming at both the preclinical and clinical levels in the years to come.
